# The relative contributions of myocardial perfusion, blood volume and extracellular volume to native T1 and native T2 at rest and during adenosine stress in normal physiology

**DOI:** 10.1186/s12968-019-0585-9

**Published:** 2019-11-25

**Authors:** Jannike Nickander, Raquel Themudo, Simon Thalén, Andreas Sigfridsson, Hui Xue, Peter Kellman, Martin Ugander

**Affiliations:** 1 Department of Clinical Physiology, Karolinska University Hospital and Karolinska Institutet, Stockholm, Sweden; 2 Department of Radiology, Karolinska University Hospital and Karolinska Institutet, Stockholm, Sweden; 3 National Heart, Lung, and Blood Institute, National Institutes of Health, Bethesda, MD USA; 4 Kolling Institute, Royal North Shore Hospital, and Northern Clinical School, Sydney Medical School, University of Sydney, Sydney, Australia

**Keywords:** Native T1, Native T2, Stress cardiovascular magnetic resonance, Quantitative perfusion, Myocardial blood volume, Extracellular volume, Mapping

## Abstract

**Background:**

Both ischemic and non-ischemic heart disease can cause disturbances in the myocardial blood volume (MBV), myocardial perfusion and the myocardial extracellular volume fraction (ECV). Recent studies suggest that native myocardial T1 mapping can detect changes in MBV during adenosine stress without the use of contrast agents. Furthermore, native T2 mapping could also potentially be used to quantify changes in myocardial perfusion and/or MBV. Therefore, the aim of this study was to explore the relative contributions of myocardial perfusion, MBV and ECV to native T1 and native T2 at rest and during adenosine stress in normal physiology.

**Methods:**

Healthy subjects (*n* = 41, 26 ± 5 years, 51% females) underwent 1.5 T cardiovascular magnetic resonance (CMR) scanning. Quantitative myocardial perfusion [ml/min/g] and MBV [%] maps were computed from first pass perfusion imaging at adenosine stress (140 microg/kg/min infusion) and rest following an intravenous contrast bolus (0.05 mmol/kg, gadobutrol). Native T1 and T2 maps were acquired before and during adenosine stress. T1 maps at rest and stress were also acquired following a 0.2 mmol/kg cumulative intravenous contrast dose, rendering rest and stress ECV maps [%]. Myocardial T1, T2, perfusion, MBV and ECV values were measured by delineating a region of interest in the midmural third of the myocardium.

**Results:**

During adenosine stress, there was an increase in myocardial native T1, native T2, perfusion, MBV, and ECV (*p* ≤ 0.001 for all). Myocardial perfusion, MBV and ECV all correlated with both native T1 and native T2, respectively (R^2^ = 0.35 to 0.61, *p* < 0.001 for all).

Multivariate linear regression revealed that ECV and perfusion together best explained the change in native T2 (ECV beta 0.21, *p* = 0.02, perfusion beta 0.66, *p* < 0.001, model R^2^ = 0.64, *p* < 0.001), and native T1 (ECV beta 0.50, *p* < 0.001, perfusion beta 0.43, *p* < 0.001, model R^2^ = 0.69, *p* < 0.001).

**Conclusions:**

Myocardial native T1, native T2, perfusion, MBV, and ECV all increase during adenosine stress. Changes in myocardial native T1 and T2 during adenosine stress in normal physiology can largely be explained by the combined changes in myocardial perfusion and ECV.

**Trial registration:**

Clinicaltrials.gov identifier NCT02723747. Registered March 16, 2016.

## Background

Coronary artery disease (CAD) and several other non-ischemic heart diseases affect the myocardial microcirculation [1]. Accurate detection of significant CAD is vital for correct treatment, as invasive revascularization improves clinical outcomes [2–4]. Global and focal reduction in myocardial blood flow (MBF) [ml/min] during adenosine stress first pass imaging with cardiovascular magnetic resonance (CMR) has a high diagnostic accuracy for detecting significant coronary stenosis [2–6]. However, MBF alone does not mirror all aspects of myocardial ischemia and the increase in myocardial oxygen demand [7–9]. Myocardial blood volume (MBV) [%] represents the total blood volume of the myocardium in the arteries, capillaries, and veins, and changes in MBV have been shown to be more sensitive than changes in MBF in the setting of elevated myocardial oxygen consumption [10, 11]. Significant coronary artery stenosis induces an increase in MBV by capillary recruitment and dilatation to supply the demanded oxygen to the cardiac myocytes. Disturbances in MBV can detect and determine the functional relevance of a significant coronary stenosis [12].

Parametric pixel-wise mapping has been developed to quantitatively measure and image the longitudinal relaxation time constant (T1) [13], and the transverse relaxation time constant (T2) [14]. Native myocardial T1 mapping has become a valuable diagnostic tool for differentiating healthy myocardium from various pathologies including acute myocardial infarction [15, 16], myocarditis [17, 18], amyloidosis [19], edema [20], Anderson-Fabry disease [21] and other non-ischemic diseases [22, 23]. Native T2 mapping allows for detection of edema in acute myocardial infarction [16, 24] and myocarditis [25], as well as estimation of area at risk [26]. Furthermore, both native myocardial T1 and T2 values depend on myocardial blood T1 and T2, respectively, due to the influence of the characteristics of the blood in the myocardium on the measured characteristics of the overall myocardium including the blood therein [27]. Native myocardial T1 mapping may be used to detect changes in MBV without the use of intravenous contrast agents [28], and potentially be used to improve ischemia detection [29, 30], which native T2 mapping also may detect [31, 32]. However, it is not clear what the changes in T1 values detected by native myocardial T1 mapping during adenosine stress signify, and if these changes apply to native T2 mapping. Recent developments in CMR allow fully automated acquisition and reconstruction of quantitative myocardial perfusion maps [ml/min/g] and MBV maps [%] [33], with an excellent agreement with clinical positron emission tomography (PET) [34]. The current study sought to elucidate the relationships between the respective parameters in normal physiology by measuring quantitative native T1, native T2, perfusion, MBV, and extracellular volume (ECV) mapping, both at rest and during adenosine stress in healthy subjects.

## Methods

### Study population

Healthy subjects (*n* = 43, 26 ± 5 years, 51% females) were included if they had no use of cardiovascular medication and had no previous history of cardiovascular symptoms or systemic disease, kidney disease, or asthma, were current non-smokers, and had a normal 12-lead electrocardiogram (ECG). The 12-lead ECG and a venous blood sample, used to determine blood hematocrit, were collected immediately prior to the CMR exam. Exclusion criteria included failed splenic switch off [35], low peak of contrast agent (low arterial input function (AIF) peak, defined as < 2 mmol/l) during first-pass perfusion imaging, and poor image quality. Ethical approval was granted for all study procedures by the local ethics committee, and all subjects provided written informed consent. The study was registered with the Clinicaltrials.gov identifier NCT02723747.

### Image acquisition

**Fig. 1:**
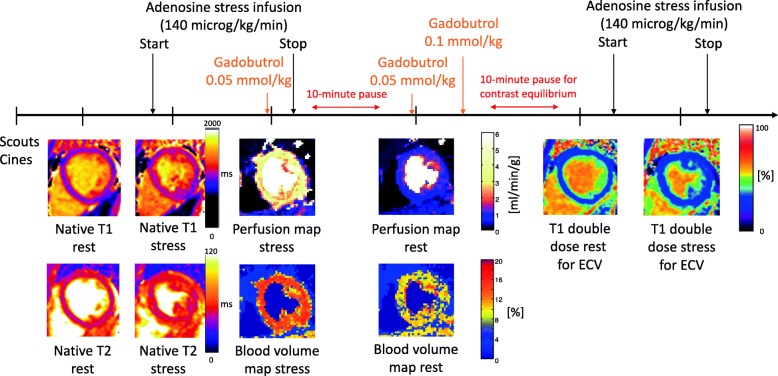
Timeline of the CMR scanning protocol. Scouts and cines were acquired first, followed by native T1 and T2 mapping at rest. Following 3 min of adenosine infusion, native T1 and T2 mapping were acquired, followed by first-pass perfusion imaging using gadobutrol (0.05 mmol/kg), which generated a perfusion map and a blood volume map. After the adenosine infusion was terminated, a 10-min pause followed to reach contrast equilibrium. First-pass perfusion images were acquired using gadobutrol (0.05 mmol/kg) at rest and an additional dose of gadobutrol (0.1 mmol/kg) was administered. Finally, post-contrast T1 map was acquired at rest, and at stress following 3 min of adenosine infusion

CMR was conducted at 1.5 T (Aera, Siemens Healthineers, Erlangen, Germany) with a phased-array eighteen-channel body matrix coil together with a spine matrix coil. All patients were examined in the supine position. Figure 1 shows a timeline of the CMR scanning protocol. First CMR scans assessing left ventricular (LV) function at rest used retrospectively ECG-gated balanced steady state free precession (bSSFP) cine imaging covering the entire LV in short-axis slices, and three long-axis slices were acquired. Typical imaging parameters included flip angle (FA) 68°, pixel size 1.4 × 1.9 mm^2^, slice thickness 8.0 mm, echo time (TE) 1.19 ms, repetition time(TR) 2.85 ms, matrix size =144 × 256, and field of view (FOV) 360 × 270 mm^2^.

Three short-axis slices (basal, midventricular, apical) were acquired during first pass perfusion imaging, both during adenosine stress (Adenosin®, Life Medical AB, Stockholm, Sweden, 140 microg/kg/min infusion) and at rest, during administration of an intravenous bolus of contrast agent (0.05 mmol/kg, gadobutrol, Gadovist®, Bayer AB, Solna, Sweden). Adenosine was administered in one cannula, while the contrast agent was administered in a separate cannula in the opposite arm. All maps were automatically generated using inline perfusion software implemented in the Gadgetron online reconstruction framework [36] and the perfusion and MBV maps were computed based on a distributed tissue exchange model [37] as previously described [33]. First-pass imaging was performed with a prototype optimized dual sequence with a fast low angle shot (FLASH) readout for AIF and bSSFP readout for myocardium. Typical imaging parameters for bSSFP single shot readout were: TE 1.04 ms, TR 2.5 ms, FA 50°, saturation delay (TS)/trigger delay (TD) 95/40 ms. Typical imaging parameters for FLASH were TE/TR 1.0/2.1 ms, FA 14°, TS/TD 100/62 ms, and common imaging parameters included bandwidth 1085 Hz/pixel, FOV 360 × 270 mm^2^, slice thickness 8.0 mm and parallel acquisition technique factor 3.

Three short axis native myocardial T1 maps (basal, midventricular, apical) were acquired using an ECG-gated modified Look-Locker inversion recovery (MOLLI) sequence [38] with a 5s(3s)3s sampling scheme (Siemens WIP 1041). The T1 maps were acquired before and during adenosine stress (140 microg/kg/min infusion). Three midventricular post-contrast T1 maps (MOLLI, 5s(3s)3s) [39] with the same slice positions as the native T1 maps, were acquired following intravenous contrast (total cumulative dose over three boluses: 0.2 mmol/kg gadobutrol), both at rest and after re-stressing with adenosine stress (140 microg/kg/min infusion) for stress ECV maps. The T1 maps were reconstructed using in-line motion correction [38], and reconstruction output included a T1 error map [40], a T1* map and the T1 map. Typical imaging parameters included bSSFP single-shot readout in end-diastole, FA 35°, pixel size 1.4 × 1.9 mm^2^, slice thickness 8.0 mm, imaging duration 167 ms, TE/TR 1.12 ms/2.7 ms, matrix size =144 × 256, FOV 360 × 270 mm^2^ and breath hold 10.7 s. Three rest and stress ECV maps were generated offline from rest pre- and post-contrast T1 maps, and stress pre- and post-contrast T1 maps, respectively, and calibrated by blood hematocrit [41], employing the formula describing the relationship between ECV and native and post-contrast T1 in the myocardium and blood including hematocrit [42].

Three myocardial native T2 maps (basal, midventricular, apical) were acquired before and during adenosine stress (140 microg/kg/min infusion). T2 mapping was performed using the Siemens MyoMaps product sequence, which is a T2-prepared sequence. The T2 map was calculated from 3 single-shot acquisitions at different T2 preparation times (0 ms, 25 ms, and 55 ms). Typical imaging parameters included TE/TR = 1.06/2.49 ms, FA 70°, pixel size 1.4 × 1.4 mm^2^, slice thickness 8.0 mm, acquisition window 700 ms, TD 483 ms, matrix size = 144 × 256, acceleration factor = 2. Inline motion registration was performed followed by a pixel-wise parametric fit to estimate the final T2 maps.

The subjects did not undergo late gadolinium enhancement (LGE) imaging as focal myocardial scar from silent myocardial infarction or myocarditis would be visible in rest ECV maps.

### Image analysis

Apical stress ECV maps were not of sufficient quality due to partial volume effects, and a sizable portion of the basal stress ECV maps were affected by the more prominent through-plane motion associated with imaging at a higher heart rate during stress. Thus, only the midventricular images were analyzed. The midventricular native T1, native T2, perfusion, MBV and ECV maps were analyzed with Segment software version 2.0 R5152 (Medviso AB, Lund, Sweden). All the respective average values were acquired by conservative manual delineation of a circumferential region of interest in the midmural third of the myocardium in the respective maps, both at rest and stress. To assess reproducibility, segmental analysis of the respective maps was performed by 3 independent observers.

LV volumes, ejection fraction and myocardial mass were quantified using the SyngoVia Software, VA30 (Siemens Healthineers), including manual corrections. Body surface area (BSA) was calculated with the Dubois formula [43]. Volumetric measurements and myocardial mass were indexed to BSA.

### Statistical analysis

Continuous variables were reported as means together with their standard deviation (SD). Ordinal variables were reported as percentages. The relationships between perfusion, MBV, ECV, heart rate, systolic blood pressure, rate pressure product and hematocrit, and native T1 and native T2, respectively, were assessed with Pearson’s linear correlation coefficient, and presented as R^2^. Significant univariate associations were entered into the multivariate linear regression analysis. Multivariate linear regression was assumed for native T1 and native T2, respectively, and was used to investigate the best explanatory parameters for native T1 and native T2, respectively, using manual stepwise linear regression modeling. By combining rest and stress absolute values in the same data set, the absolute changes in all parameters were investigated by linear regression. Mean values were compared by using the paired or unpaired t-test as appropriate in normally distributed data, as decided by the Kolmogorov-Smirnov test. Interobserver variability was assessed by intraclass correlation. Intraobserver variability was calculated as the average difference of two measurements divided by their mean, and expressed as a percentage. Statistical analysis was performed using Microsoft Excel® (Microsoft, Redmond, Washington, USA) and SPSS Statistics® (Statistical Package for the Social Sciences (SSPS), International Business Machines, Inc., Armonk, New York, USA). The significance level in all statistical analyses was defined as *p* < 0.05.

## Results

### Study population

In total, two of the healthy subjects were excluded (failed contrast injection, *n* = 1, and low peak AIF, *n* = 1). Table 1 shows baseline characteristics of the study cohort (*n* = 41). Stress ECV images could not be processed in 4 cases because pre and post T1 maps were mistakenly acquired with different FOV, and these cases were excluded from analysis of ECV. No subjects had any evidence of any focal myocardial scarring in any of the three short-axis maps. No subject was excluded due to poor image quality.

**Table 1:** Baseline subject characteristics

Characteristic	*n* = 41
Female sex, *n* (%)	21 (51)
Age, years	26 ± 5
Height, cm	175 ± 9
Weight, kg	71 ± 11
BSA, m^2^	1.9 ± 0.2
LVEDV, ml	176 ± 36
LVEDVI, ml/m^2^	94 ± 14
LVESV, ml	72 ± 17
LVESVI, ml/m^2^	39 ± 7
LVSV, ml	104 ± 22
LVSVI, ml/m^2^	56 ± 9
LVEF, %	59 ± 4
LVM, g	119 ± 28
LVMI, g/m^2^	63 ± 11
Rest ECV, %	27 ± 3
Rest heart rate, bpm	71 ± 12
Stress heart rate, bpm	95 ± 16
Rest systolic blood pressure, mmHg	109 ± 11
Stress systolic blood pressure, mmHg	112 ± 12
Rest rate pressure product, bpm*mmHg	7815 ± 1893
Stress rate pressure product, bpm*mmHg	10,609 ± 2451
Change in rate pressure product, %	38 ± 28

Data presented as mean ± SD. Abbreviations: *BSA* body surface area, *ECV* extracellular volume, *LVEDV* left ventricular end diastolic volume, *LVESV* left ventricular end systolic volume, *LVSV* left ventricular stroke volume, *LVEF* ejection fraction, *LVM* left ventricular mass, *I* signifies indexed to BSA

### Reproducibility

Inter- and intraobserver variability were low, and are presented in Additional file 1.

### Myocardial T1, T2, MBV, perfusion and ECV during adenosine stress

**Fig. 2:**
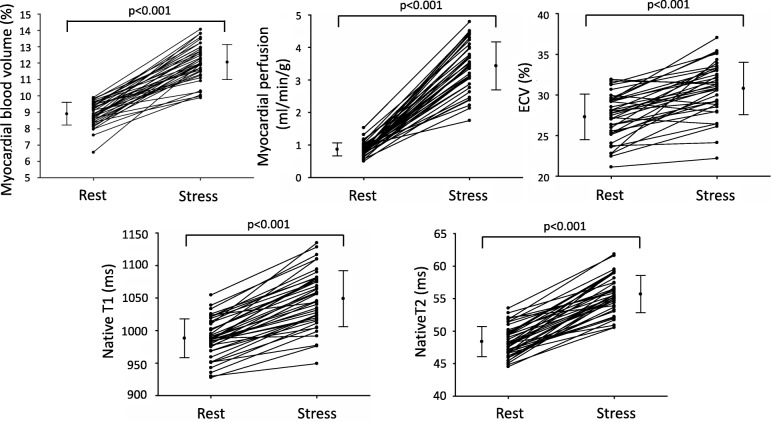
The effect of adenosine stress on native T1, native T2, myocardial perfusion, MBV and ECV. All mean values of the respective parameters increased with adenosine stress. Three healthy subjects decreased in ECV during adenosine stress, which is likely due to low measurement precision affected by heart rate at stress

From rest to stress there was an increase in native T1 (988 ± 30 vs 1049 ± 43 ms, *p* < 0.001), native T2 (48 ± 2 vs 56 ± 3 ms, *p* < 0.001), MBV (9.0 ± 0.7 vs 12.1 ± 1.1%, *p* < 0.001), myocardial perfusion (0.9 ± 0.2 vs 3.4 ± 0.7 ml/min/g, p < 0.001), and ECV (27.3 ± 2.8 vs 30.9 ± 3.2%, *p* < 0.001), see Fig. 2. Segmental values are presented in Additional file 1.

### The change in MBV, perfusion and ECV in relation to the change in myocardial T1 and T2

**Fig. 3:**
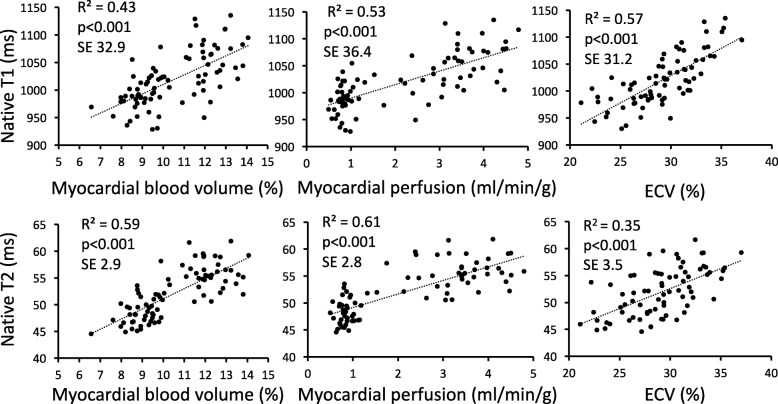
Linear correlation between native T1 or native T2 and the respective parameters. Native T1 correlated with myocardial blood volume (MBV), myocardial perfusion and myocardial extracellular volume (ECV) (R^2^ range 0.41–0.57). Native T2 correlated with MBV, myocardial perfusion and ECV (R^2^ range 0.35–0.61)

The relationships between myocardial T1, T2, MBV, perfusion and ECV assessed individually at rest and stress, respectively, are presented in Additional file 1. Heart rate, systolic blood pressure, rate pressure product and hematocrit did not correlate with native T1 and native T2 at stress. Thus, these parameters were not included in the multivariate linear regression analysis. In combined analysis of pooled rest and stress data, myocardial perfusion, MBV and ECV all correlated with both native T1 and native T2, respectively (R^2^ = 0.35 to 0.61, *p* < 0.001 for all), Fig. 3. Furthermore, multivariate linear regression showed that ECV and perfusion together best explained the change in native T2 (ECV beta 0.21, *p* = 0.02; perfusion beta 0.66, *p* < 0.001, model R^2^ = 0.64, *p* < 0.001), and change in native T1 (ECV beta 0.50, *p* < 0.001; perfusion beta 0.43, *p* < 0.001, model R^2^ = 0.69, *p* < 0.001), Table 2.

**Table 2:** Multivariate linear regression for the relative contribution to native T1 and native T2

Multivariate linear regression	Myocardial perfusion beta, *p*-value	Extracellular volume beta, p-value	Model R^2^, *p*-value
Native T1	0.50, *p* < 0.001	0.43, *p* < 0.001	0.69, *p* < 0.001
Native T2	0.21, *p* = 0.02	0.66, *p* < 0.001	0.64, *p* < 0.001

## Discussion

We demonstrate that native myocardial T1, native myocardial T2, myocardial perfusion, MBV, and ECV all increase in response to adenosine stress in healthy subjects. The changes in native myocardial T1 and T2 values are best explained by the combined changes in myocardial perfusion and ECV, thus supporting the notion that native myocardial T1 mapping, and T2 mapping indeed reflect the increase of blood in the myocardium during adenosine stress, and illustrating the mechanism behind the potential to use native T1 or T2 to detect CAD without the need for an intravenous contrast agent.

### Native mapping for quantifying myocardial blood volume and flow

**Fig. 4:**
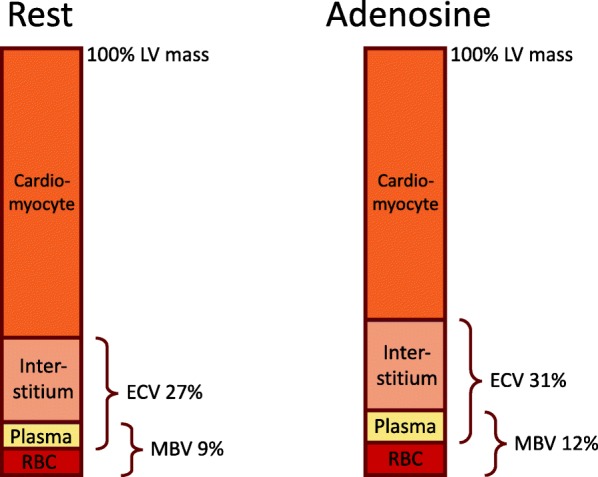
Schematic illustration summarizing the relative sizes of different myocardial compartments and their change between rest and adenosine stress. The bars, representing 100% of the left ventricular (LV) mass, illustrate that myocardial blood volume (MBV) is approximately 9% at rest, and consists of plasma and red blood cells (RBC). The myocardial extracellular volume (ECV) is approximately 27% at rest, and consists of plasma and interstitium. The rest of the LV mass consists of cardiomyocytes. At stress, in terms of percentage of the LV mass, MBV increases to 12%, likely due to a balanced increase in both plasma and RBC. Myocardial ECV also increases to 31%, likely reflecting the increase in plasma. It is not known if the absolute LV mass increases during stress. Considering that compartments other than the cardiomyocytes (interstitium, blood) do increase during stress, it is likely that the absolute volume of cardiomyocytes stays the same, and the total LV mass increases slightly during stress. This can explain why, in this illustration, the cardiomyocyte compartment appears to have decreased in size, when expressed as a percentage of total LV mass. Furthermore, it is not known if the relationship between the increase in the RBC and plasma components of MBV is 1:1, since most of the increase lies within capillaries, which may have a different hematocrit compared to the blood in larger vessels

In the current study, we quantified myocardial perfusion and MBV using first-pass contrast-enhanced CMR. Several approaches have been proposed for quantifying myocardial perfusion and MBV. PET [44] and myocardial contrast echocardiography (MCE) [45] have been used to quantify both myocardial perfusion and MBV, albeit with different methodologies, respectively. In CMR, the most widely used technique for quantification of myocardial perfusion and MBV is first-pass perfusion imaging [9, 46, 47]. A limitation with PET is the use of ionizing radiation, and MCE has substantial operator dependency. While CMR first-pass perfusion requires the use of a gadolinium-based contrast agent, native T1 and T2 mapping do not require such agents. T1 mapping quantifies the T1 relaxation time, and the T1 is expected to be prolonged by increased myocardial water content [17, 18, 20] including the increase in water associated with an increased myocardial blood volume. By this reasoning, an increase in myocardial blood would lead to an increase in myocardial T1. This should be true for native T2 mapping as well, since T2 mapping quantifies the T2 relaxation time, which also is influenced by the increase in myocardial water content [14]. However, the relationship between vasculature, blood flow and blood volume has not been completely elucidated [7–9], and in vivo quantification in humans complicates the use of conventional validation methods, such as the radiolabeled ^99m^Tc-Red-Blood-Cell method [7, 48]. Furthermore, while adenosine does not increase myocardial oxygen demand, the vasodilator stress induced by adenosine mimics the physiological vasodilation during exercise. In this multiparametric mapping study we used first-pass myocardial perfusion maps, MBV maps, and ECV maps. It is not fully known how myocardial perfusion and MBV contribute to the increase in native T1 and T2. However, this study supports the notion that the increase in myocardial ECV is due to the increase in intramyocardial plasma during adenosine stress, which correlates with the increase in intramyocardial blood detected by MBV mapping. This is summarized and illustrated schematically in Fig. 4.

### Myocardial blood volume or myocardial perfusion

A study cohort of healthy subjects with low measurement variability may not fully elucidate the relationships between the ECV, MBV and perfusion with native T1 and native T2 at rest. Therefore, a multivariate linear regression analysis of the changes in the parameters was employed by investigating rest and stress in a combined fashion. This introduced a variability in native T1 and T2 necessary to evaluate the relevant relationships. The multivariate analysis showed that the combined changes in myocardial perfusion and ECV best explain the increase in native T1 and T2 mapping in response to adenosine stress. This is furthered supported by the finding in the univariate analysis for native T1 and native T2 at rest and stress, respectively. The analyses indicate that ECV was the main contributor to these measures, see Additional file 1. However, the relationship between vasculature, blood flow and blood volume has not been fully elucidated. It is still not completely determined if the increase in native T1 and T2 is exclusively due to the increase in myocardial perfusion. The newly developed MBV maps used in this study might have slightly lower measurement precision compared to ECV maps, which could be a possible explanation for the fact that MBV was not significant in the multivariate analysis. Extensive work has been done to increase measurement precision and accuracy with native T1 and ECV mapping [38, 40, 41, 49–52], whereas the MBV maps are new and have not yet been validated using independent reference standards. Furthermore, the intra-subject repeatability needs to be investigated for these newly developed MBV maps, since the biological variability in MBV is still unknown. Drawing upon the results from intra-subject repeatability of myocardial perfusion maps [53], there should be a good repeatability for the average values used in the current study. Other factors that potentially could affect myocardial perfusion quantification include choice of vasodilator, choice of contrast agent, vendor, pulse sequence approach, and normal biological variability, all of which merit further investigation. In our own data at 1.5 T and 3 T, we have not seen any difference between rest and stress perfusion values [unpublished data currently submitted for publication], however it would be of further interest to analyze data from different sites in order to establish robust normal values for this newly developed quantitative perfusion sequence. Notably, it is possible that native mapping cannot differentiate between MBV and myocardial perfusion, and that there is a need for a contrast agent to differentiate between MBV and myocardial perfusion. While MBV and myocardial perfusion are closely linked, the results are of interest given that the currently proposed physiological explanation is that the change in native T1 is primarily dependent upon the changes in MBV, which this study cannot exclusively determine. The results of this study are important to add to the current body of knowledge regarding stress native mapping. The most important finding of this study is that both native T1 and T2 indeed increase in response to adenosine, and are associated with MBV, ECV, and myocardial perfusion. This finding supports the hypothesis that native T1 mapping can depict the coronary flow reserve as suggested in a study where native T1 response during adenosine was blunted in patients with aortic stenosis, but normalized following intervention [30]. This is also supported by the finding that native T1 response to adenosine stress was blunted in areas of chronic infarction verified by LGE [29], and a study that found that the native T1 response to adenosine is blunted in patients with diabetes type 2 [54]. The findings in this study suggest that T2 mapping may also be used to depict the myocardial perfusion reserve during adenosine stress.

### MOLLI sequences in native T1 stress

In the current study the MOLLI 5s(3s)3s sequence was used due to the clinical routine at the time of the study. MOLLI sequences can be heart rate dependent due to two major factors; the time between inversion, and the SSFP readout influence on each inversion recovery. By setting the time between inversions in seconds instead of heart beats as in the MOLLI 5(3)3 protocol, heart rate dependency is mitigated [39], however it introduces fixed breath holds, which by design are longer compared to the MOLLI 5(3)3 protocol during stress conditions. Another approach is to use the shortened MOLLI (ShMOLLI) sequence [55], that reduces heart rate dependency, however introduces a loss of precision associated with discarding data [39]. It is currently unknown how MOLLI 5(3)3, MOLLI 5s(3s)3s and ShMOLLI compares to each other in terms of accuracy and precision in native T1 stress imaging. A study where these different protocols are compared head to head would be of value in order to determine the optimal stress native T1 mapping technique moving forward. Factors such as motion artifacts due to poor breath hold, and loss of precision are, of course, of importance. However, as has recently been suggested, clinical data will ultimately determine the best protocol [56].

### Clinical outlook

A recent study suggests that stress native T1 mapping can be used to differentiate between obstructive epicardial disease and microvascular disease [57], the mechanisms of which have in part been elucidated by the findings in the current mechanistic study. Native T1 and T2 mapping correlate closely with myocardial perfusion, MBV and ECV during adenosine stress, which highlights the physiological foundation for native T1 or native T2 mapping to be used to identify myocardial ischemia. In this mechanistic study, the midmural third of the myocardium was analyzed in order to mitigate factors such as partial volume effects and blood pool contamination. In a clinical setting this erosion may affect the detection of CAD as myocardial ischemia and cell death appears initially in the subendocardium in accordance with the wavefront phenomenon [58]. Other studies have successfully identified obstructive CAD and microvascular disease using adenosine stress native T1 mapping [29, 57, 59]. Notably, the current study did not seek to evaluate whether or not T1 or T2 mapping may be used to diagnostically differentiate between epicardial and microvascular disease, but rather to elucidate if there is a physiological foundation for diagnostic use of native T1 or T2 mapping in a coronary disease setting by interrogating the physiological mechanisms in normal physiology. Furthermore, it would be of value to investigate regional variability in native T1 and native T2 mapping during stress in patients to see if microvascular dysfunction can be identified using the approach with regional myocardial perfusion reserve using gadolinium-based contrast [59]. This could potentially remove contrast agents from the assessment of myocardial ischemia with CMR.

## Limitations

This study was conducted in a cohort of healthy subjects free of known cardiovascular disease, and therefore there is a need for larger scale studies to confirm the findings in older subject and patients with CAD. Furthermore, some of the subjecs had a high level of fitness, which is reflected in the slightly higher LV volumes and slightly lower LV ejection fraction, which is in agreement with previously published values in athletes [60]. The findings were only quantified in one midventricular short-axis slice, due to limited quality of basal and apical stress ECV maps, which reflects one region of the heart and not the entire LV. Basal and apical stress ECV maps were of limited image quality. Since this is a mechanistic study, one midventricular slice was deemed adequate to address the aims of the study. As CAD is primarily a focal disease involving one vessel, it’s likely that three short axis slices can capture disturbances in MBV and myocardial perfusion better than one midventricular short-axis slice. Therefore, the applicability of native T1 and T2 mapping needs to be investigated in a clinical population. The use of ECV mapping as a validation method could have potentially introduced minor measurement errors, due to subtraction of the two separate native and post-contrast T1 maps necessary to generate one ECV map, and the fact that the 5s(3s)3s protocol was used for post-contrast T1 mapping due to clinical routines. The 5s(3s)3s protocol has a slightly lower accuracy and precision in lower T1 values compared to a 4s(1s)3s(1s)2s protocol [39], which could theoretically lead to a small but likely negligible overestimation of ECV. Furthermore, stress ECV maps are dependent upon two separate stress imaging sessions, however it has been shown that there is no difference in repeated intra-study quantification of global perfusion at both rest and stress [53]. All maps were acquired in the same order, which potentially could introduce systematic bias in the native mapping results. However, drawing upon the excellent intrastudy reproducibility of global perfusion at both rest and stress [53], there is likely no physiological bias related to the order of imaging during stress.

The T2 mapping technique used in this work was based on a T2 prepared sequence, which may introduce heart rate sensitivity, off-resonance, and T1 dependencies in the quantification.

## Conclusions

Myocardial native T1, native T2, perfusion, MBV, and ECV all increase during adenosine stress in healthy subjects. Changes in myocardial native T1 and T2 during adenosine stress in normal physiology can largely be explained by the combined changes in myocardial perfusion and ECV.

## Supplementary information

**Additional file 1.** Reproducibility, segmental values and respective linear regression. Data include tables on intra- and interobserver variability, segmental values of the respetive maps, and linear regression at rest alone and stress alone.

## Data Availability

The data that supports the findings of this study is available from corresponding author upon reasonable request.

## Abbreviations

AIF: Arterial input function
BSA: Body surface area
bSSFP: Balance steady state free precession
CAD: Coronary artery disease
CMR: Cardiovascular magnetic resonance
ECG: Electrocardiogram
ECV: Extracellular volume fraction
FA: Flip angle
FLASH: Fast low angle shot
FOV: Field of view
LGE: Late gadolinium enhancement
LV: Left ventricle/left ventricular
MBF: Myocardial blood flow
MBV: Myocardial blood volume
MCE: Myocardial contrast echocardiography
MOLLI: Modified Look-Locker inversion recovery
PET: Positron emission tomography
SD: Standard deviation
ShMOLLI: Shortened modified Look-Locker inversion recovery
TD: Trigger delay
TE: Echo time
TR: Repetition time
TS: Saturation delay
