# Supplementary material for: The relative contributions of myocardial perfusion, blood volume and extracellular volume to native T1 and native T2 at rest and during adenosine stress in normal physiology
Source: J Cardiovasc Magn Reson. 2019 Nov 25;21:73. doi: 10.1186/s12968-019-0585-9 (PMC6876099; doi:10.1186/s12968-019-0585-9)
Supplement: Supplementary file 1 — Additional file 1. Reproducibility, segmental values and respective linear regression. Data include tables on intra- and interobserver variability, segmental values of the respetive maps, and linear regression at rest alone and stress alone. [file 12968_2019_585_MOESM1_ESM.docx]

## Additional file 1

## Reproducibility

Table 1 shows inter- and intraobserver variability of the global values of all maps at rest and stress respectively.

**Table 1. Inter- and intraobserver variability**

|  | Interobserver,  ICC | Intraobserver, mean difference (%) |
| --- | --- | --- |
| Native T1 rest | 0.95, p<0.001 | 2.3±1.5 |
| Native T1 stress | 0.95, p<0.001 | 1.7±1.0 |
| Native T2 rest | 0.87, p<0.001 | 1.8±4.7 |
| Native T2 stress | 0.82, p<0.001 | 0.2±4.6 |
| MBV rest | 0.90, p<0.001 | 3.2±4.8 |
| MBV stress | 0.87, p<0.001 | 4.6±5.3 |
| ECV rest | 0.97, p<0.001 | 1.6±0.7 |
| ECV stress | 0.95, p<0.001 | 1.6±0.9 |
| Perfusion rest | 0.95, p<0.001 | 1.3±6.0 |
| Perfusion stress | 0.95, p<0.001 | 1.2±4.7 |

Abbreviations: ECV – extracellular volume, ICC – intraclass correlation, MBV – myocardial blood volume

## Segmental values

Table 2 shows segmental values for native T1, native T2, MBV, ECV and perfusion maps at rest and stress respectively.

**Table 2. Segmental values from one midventricular short-axis image.**

|  | Anterior | Antero-  septal | Infero-  septal | Inferior | Infero-  lateral | Antero-  lateral |
| --- | --- | --- | --- | --- | --- | --- |
| Native T1  rest, ms | 976±63 | 1000±31 | 1010±38 | 981±35 | 968±43 | 982±43 |
| Native T1  stress, ms | 1062±64 | 1071±58 | 1067±56 | 1053±56 | 1049±60 | 1051±52 |
| Native T2  rest, ms | 50±4 | 49±3 | 50±3 | 48±4 | 48±4 | 51±4 |
| Native T2  stress, ms | 57±5 | 59±5 | 58±4 | 54±7 | 56±5 | 55±5 |
| MBV  rest, % | 8.9±1.2 | 9.4±1.1 | 9.4±1.1 | 9.0±1.2 | 8.8±1.4 | 8.8±1.4 |
| MBV  stress, % | 12.2±1.9 | 12.9±1.4 | 11.1±1.4 | 12.1±1.7 | 12.0±2.0 | 12.0±1.8 |
| ECV  rest, % | 27 ± 4 | 28 ± 5 | 29 ± 4 | 26 ± 3 | 26 ± 4 | 27 ± 4 |
| ECV  stress, % | 31 ± 4 | 32 ± 4 | 31 ± 3 | 30 ± 4 | 29 ± 4 | 30 ± 4 |
| Perfusion  rest, ml/min/g | 0.8±0.2 | 0.8±0.2 | 0.8±0.2 | 0.8±0.2 | 0.7±0.2 | 0.8±0.2 |
| Perfusion  stress, ml/min/g | 3.6±0.8 | 3.4±0.8 | 3.4±0.8 | 3.4±0.8 | 3.4±0.8 | 3.5±0.8 |

Abbreviations: ECV – extracellular volume, MBV – myocardial blood volume

## Relationships between myocardial T1, T2, MBV, perfusion and ECV

Table 3 shows that native T1 values at rest correlated with myocardial perfusion and ECV at rest but not with MBV at rest, and native T1 values at stress correlated with myocardial perfusion and ECV at stress. By comparison, native T2 values at rest correlated with ECV and MBV at rest but not with myocardial perfusion at rest, while native T2 values at stress did not correlate with myocardial perfusion, MBV and ECV at stress. Multivariate analysis showed that ECV was the main contributor to native T1 both at rest alone (ECV beta 0.62, p<0.001, model R^2^=0.38, p<0.001) and stress alone (ECV beta 0.67, p<0.001, model R^2^=0.45, p<0.001). The main contributor to native T2 at rest alone was ECV (ECV beta 0.45, p<0.01, model R^2^=0.21, p<0.01). At stress alone, there was no significant contributor, however there was a trend towards ECV (ECV beta 0.28, p=0.09, model R^2^=0.08, p=0.09).

**Table 3. Summary of linear regression at rest alone and stress alone, respectively, for the relative contribution to native T1 and native T2.**

| Linear regression | Myocardial Perfusion  R^2^, p-value | MBV  R^2^, p-value | ECV  R^2^, p-value |
| --- | --- | --- | --- |
| Native T1 rest | 0.12, p=0.03 | 0.05, p=0.15 | 0.38, p<0.001 |
| Native T1 stress | 0.28, p<0.001 | 0.08, p=0.08 | 0.45, p<0.001 |
| Native T2 rest | 0.07, p=0.10 | 0.11, p=0.04 | 0.21, p=0.01 |
| Native T2 stress | 0.01, p=0.46 | 0.01, p=0.46 | 0.08, p=0.09 |

Abbreviations: ECV – extracellular volume; MBV – myocardial blood volume
